# Violence and aggression in psychiatric inpatient care in Sweden: a critical incident technique analysis of staff descriptions

**DOI:** 10.1186/s12913-020-05239-w

**Published:** 2020-04-26

**Authors:** Veikko Pelto-Piri, Lars-Erik Warg, Lars Kjellin

**Affiliations:** 1 University Health Care Research Center, Faculty of Medicine and Health, Örebro University, Örebro, Sweden; 2 School of Law, Psychology and Social Work, Örebro University, Örebro, Sweden

**Keywords:** Violence, Staff, Psychiatry, Inpatient, Critical incident technique, Qualitative content analysis, Prevention

## Abstract

**Background:**

Violence towards staff working in psychiatric inpatient care is a serious problem. The aim of the present study was to explore staff perspectives of serious violent incidents involving psychiatric inpatients through the following research questions: Which factors contributed to violent incidents, according to staff? How do staff describe their actions and experiences during and after violent incidents?

**Methods:**

We collected data via a questionnaire with open-ended questions, and captured 283 incidents reported by 181 staff members from 10 inpatient psychiatric wards in four different regions. We used the Critical Incident Technique to analyse the material. Our structural analysis started by structuring extracts from the critical incidents into *descriptions*, which were grouped into three chronological units of analyses: *before the incident*, *during the incident* and *after the incident.* Thereafter, we categorised all descriptions into *subcategories*, *categories* and *main areas.*

**Results:**

Staff members often attributed aggression and violence to internal patient factors rather than situational/relational or organisational factors. The descriptions of violent acts included verbal threats, serious assault and death threats. In addition to coercive measures and removal of patients from the ward, staff often dealt with these incidents using other active measures rather than passive defence or de-escalation. The main effects of violent incidents on staff were psychological and emotional. After violent incidents, staff had to continue caring for patients, and colleagues provided support. Support from managers was reported more rarely and staff expressed some dissatisfaction with the management.

**Conclusions:**

As a primary prevention effort, it is important to raise awareness that external factors (organisational, situational and relational) are important causes of violence and may be easier to modify than internal patient factors. A secondary prevention approach could be to improve staff competence in the use of de-escalation techniques. An important tertiary prevention measure would be for management to follow up with staff regularly after violent incidents and to increase psychological support in such situations.

## Background

Violence is a serious problem in the psychiatric inpatient care environment [1]. Violence towards staff affects their physical and psychological health and can result in stress, absenteeism, and resignations [2–6]. In our previous study from 2018, 47% of staff members working in psychiatric inpatient care in Sweden experienced violence during the 6 months prior to the study [7]. To implement strategies aimed at preventing violent incidences, we need to understand the different contexts in which staff members experience violence and their perceptions of the underlying causes [5, 8].

Primary violence prevention in psychiatric inpatient care aims to create a ward climate that minimizes the risk of violence [9]; to achieve this, we need to understand the determinants of violence. Background factors causing violent behaviour can be sorted into three conceptual models: internal, external and situational/relational [8, 10]. The *internal model* deals with factors emanating from the patient. Severe mental disorders, especially schizophrenia and other types of psychoses, are often put forward as major predictors of inpatient aggression [11]. Substance abuse, a history of violence and demographic factors are also important when in conjunction with a mental disorder [12]. The *external model* focuses on environmental factors in psychiatry to explain aggressive and violent behaviour, such as ward size and spaciousness, the level of surveillance by staff, the professional experience of the nurses and the preventive strategies in place [13–15]. The *situational/relational model* focuses on relationships at the ward. Negative staff–patient interactions often lead to patient aggression and coercive measures [10, 13, 16, 17]. Violent incidents can be prevented if staff are more engaged in their work, trust is created between staff and patients, and positive social activities are instituted in the ward [18–21]. An increasingly popular model in psychiatric settings is the Safewards model, a comprehensive model that includes internal factors as well as external and situational/relational factors [22]. The originating factors in the model are the patient community, patient characteristics, regulatory framework, staff team, physical environment and stressors from outside the hospital. Problems associated with any of these factors can give rise to flashpoints, triggering conflicts and leading to containment. The Safewards model also suggests ways that staff can prevent such flashpoints from arising.

In terms of secondary prevention, the Safewards model suggests how flashpoints can be de-escalated [22]. To avoid restrictive practices, staff need to recognise the early signs that can lead to aggression and use calming and de-escalation techniques [23]. If restrictive preventive measures like body/property searches, seclusion or rapid tranquillisation are deemed necessary, they should be exercised with caution and with respect for the dignity of the patient [23, 24]. Research and subsequent guidelines stress that management should implement strategies and ward policies to address problems with violence, preferably through de-escalation techniques [9, 23, 25, 26]. Restrictive practices should be avoided as they can be dangerous; findings indicate that they can result in post-traumatic stress, delayed recovery, injury and even death [27–29].

When dealing with the aftermath of a violent incident, guidelines stress that management should provide immediate support. Such tertiary prevention interventions can include medical treatment, debriefing or counselling, and can be designed for victims, perpetrators, witnesses and all staff affected by the incident [9, 22, 30]. Many nurses who have experienced verbal and physical violence have experienced increased job stress [31]. The prevalence of post-traumatic stress disorder (PTSD) in psychiatric nurses is around 10% and is directly related to the high rate of assault by patients [32, 33].

To effectively implement prevention strategies, we need to know what staff think about the situation when incidents occur. Despite some recent studies [34–36], there remains a lack of research on staff and patient perspectives on violence and aggression in mental health, particularly across a variety of settings. In the present study, our aim was to explore staff perspectives on serious violent incidents that they experienced during care of psychiatric inpatients through the following research questions: Which factors contributed to violent incidents, according to staff? How do staff describe their actions and experiences during and after the violent incidents?

## Methods

### Design

The purpose of the study was to obtain rich descriptions of staff actions and experiences relating to violent incidents in different settings. To do this effectively, we collected questionnaire data according to the Critical Incident Technique (CIT) [37]. The critical incident descriptions made by staff covered a timeline of events taking place, before, during and after a violent incident [38–40] to match the primary, secondary and tertiary prevention approaches, respectively. We used structural analysis [37, 38] and present quantitative frequency counts for the subcategories, a combination of methodologies commonly used in CIT studies [41, 42].

### Settings and participants

Study participants were the entire population of staff in 10 inpatient psychiatric clinics, situated in four different regions of central Sweden. There were three different types of unit: six general psychiatric wards, three forensic psychiatric inpatient units and one psychiatric addiction centre. The length of hospital stay was often long-term in the forensic psychiatric wards and mostly short-term in the other wards. Substance users were found in all the wards, but the psychiatric addiction unit had special competence to care for patients with co-occurring addiction and psychiatric problems. For inclusion in the study, the staff had to be working at the ward during the time of the study and be dealing with patients on an everyday basis.

### Materials

The questions were open-ended and required the staff to describe in writing the two most-violent incidents that they had experienced during the previous 2 years. For each of these incidents, the first question asked for a brief description of the actual incident. This was followed by six questions expanding on the description of the incident. These questions were constructed on the basis of a questionnaire used by Hensing et al. [43] and adapted for violent incidents: (1) What happened before the incident? (2) Are there factors further back in time that could explain the incident? (3) What happened during the incident? (4) What happened after the incident? (5) What kind of consequences did the incident have (for you and others)? (6) What kind of measures did you (and others) take to deal with the problems caused by the incident?

### Data collection

Data were collected by a self-administrated questionnaire during spring 2014 and autumn 2015. The full questionnaire included items about the psychosocial work environment and about violent incidents as presented above (further information on the questionnaire can be found in our open-access article [7]). The questionnaire, along with a return envelope and a cover letter describing the purpose of the study, was distributed to 1044 members of staff. This was done by mail in three regions; in one region, a contact person at the ward, often a staff nurse, was responsible for distributing the questionnaires to the staff. Each questionnaire was labelled with a code number, and the code key was stored in a safe. A reminder was sent by mail to those who did not return the questionnaires within 3 weeks. After two more weeks, an additional reminder was sent, if necessary. In all, 443 questionnaires were returned and, of these, 181 reported critical incidents (CIs). Thus, 262 returned questionnaires did not provide any CI descriptions. Of the completed questionnaires, some were restricted to a description of only one CI, others were too vague to code, and some were general and did not refer to any specific incident. Ultimately, 283 CIs were included in the analysis: 203 were from staff who described two CIs and 80 were from staff who described one CI.

### Analysis and interpretation

The analysis began with all authors carefully reading through all of the 283 CI events on several occasions to familiarise themselves with the content and variety. Answers to the six questions described above were grouped into three chronological units of analysis: *before the incident*, *during the incident* and *after the incident.* In line with CIT tradition, the incidents were analysed with structural analysis [37–39], which is similar to qualitative content analysis with an inductive approach [44]. The structural analysis started by summarising the CIs and structuring them into *descriptions*. We then assessed the similarities and differences between the descriptions in the labelling and classifying process [38, 39]: incidents with similar descriptions were grouped into *subcategories*. These subcategories were then merged into *categories* that describe the general character of the subcategories. Finally, categories were merged into *main areas* to describe the overall content of the material. We also present quantitative results regarding the number of descriptions in the different subcategories. All of the data were available during the analysis, enabling us to continuously check and compare our categorisations with the complete staff descriptions. All categorisation and decisions were made with consensus from at least two authors; in the case of difficulties with interpretation, assistance from the third author was sought. All important decisions were taken jointly by all three authors.

## Results

The results are presented according to the chronological units of analysis: *before the incident*, *during the incident* and *after the incident*. Each unit is divided into main areas, categories and subcategories. We have provided one quotation as an example for each main area, but quotations from all subcategories are provided in the extended tables (see Additional file).

### Before the incident

Patient refuses to take the medicine. Sitting beside patient and talking about the importance of taking the medicine. Patient becomes aggressive and refuses to listen.

Table 1 shows the categorisation of factors *before* the incident that staff considered important for explaining what happened during the incident. There are three main areas, four categories and 25 subcategories.

**Table 1:** Categorisation of staff descriptions of important factors *before* the 283 incidents

Subcategory	Category	Main area
Psychiatric diagnosis/symptoms/conditions (106)	Patient traits and states	Internal patient factors
Disruptive/violent/impulsive behaviour (68)		
Substance abuse (60)		
Scared/anxious/frustrated/suspicious (34)		
Sudden threatening/violent behaviour (34)		
Intellectual disability/dementia/brain injury (10)		
Approaching staff suddenly and unpredictably (8)		
Foreign background (8)		
Self-harm/suicide attempt (7)		
Inappropriate sexual or misogynistic behaviour (5)		
Historic sexual abuse of patient (1)		
Serious somatic illness (1)		
Special and rare circumstances (5)		
Organisational problems/inadequate handling (29)	Characteristics of organisation and staff	External factors
Change of shift/staff busy (7)		
Scared/anxious/inexperienced staff (6)		
Staff preparing to intervene (1)		
Denied request/unwanted news (58)	Unwanted decision or information	Situational and relational factors
Medicine denied or enforced (31)		
Medication of patients (26)	Measures taken impacting the patient	
Patient about to be admitted/discharged (15)		
Patient in doctor or staff consultation (13)		
Police intervention (11)		
Patient supervised/secluded (7)		
Patient (about to be) mechanically restrained (6)		

The figures in parentheses indicate the number of descriptions

### Internal patient factors

This main area consists of one category labelled *patient traits and states.* The most frequent subcategories were psychiatric diagnoses (*n* = 106), disruptive behaviour (*n* = 68) and substance abuse (*n* = 60). Patients were often described, for instance, as having difficulties mastering their impulsive behaviour or paranoid beliefs. Staff experienced that patients with long-term drug addiction might be more likely to solve problems by resorting to violence. In ten of the descriptions, intellectual disability, dementia or brain injury was mentioned. These patients were described as difficult to handle since they could be unpredictable, and staff claimed that they lacked the competence to deal with their disabilities. Patients who were scared, anxious or frustrated (*n* = 34), as well as those showing sudden threatening or violent behaviour (*n* = 34) were often seen in relation to their diagnosis or disability*.* In eight descriptions, a foreign background was noted as problematic because the patients lacked an understanding of Swedish or came from countries at war. There were also descriptions of patients who demonstrated inappropriate sexual or misogynistic behaviour towards female staff (*n* = 5) and one description of a sexually abused patient who was difficult to deal with because of these experiences.

### External factors

The category *characteristics of organisation and staff* comprises external factors that, according to the CI descriptions, could precede violence in a ward, e.g. when there was shift change or when the staff were busy (*n* = 7), or if there were organisational problems and inadequate handling (*n* = 29), such as not keeping promises to the patients. Some of the CI descriptions indicated that scared, anxious or inexperienced staff could trigger violent behaviour among patients (*n* = 6).

### Situational and relational factors

We identified two categories of situational and relational factors. Preceding violent incidents, *unwanted decisions/information* often triggered the patient. One such trigger was patients being denied medicine or coerced to take it (*n* = 31). Other triggers included patients receiving bad news or having a request denied (*n* = 58), for example, being denied a leave of absence, personal equipment not being allowed at the ward, not getting more coffee during the night, not being allowed to leave part of the ward or not being able to have a driving licence owing to drug/alcohol abuse. The category *measures taken impacting the patient* include six subcategories, including medication of the patient (*n* = 26), patient about to be admitted/discharged (*n* = 15), patient in doctor or staff consultation (*n* = 13) and police intervention (*n* = 11).

### During the incident

A patient kicked me in the groin and spat on my face and then threatened to kill me and my children.

Table 2 shows the categorisation of accounts by staff members describing what happened *during* the two most aggressive or violent incidents they experienced over the previous 2 years. The CI descriptions are divided into two main areas, five categories and 24 subcategories.

**Table 2:** Categorisation of staff descriptions of what happened *during* the 283 incidents

Subcategory (number of descriptions)	Category	Main area
Aggression against property (45)	Material damage	Details of violent and aggressive acts and situations
Verbal threats/provocation/aggression (89)	Violent patient acts towards staff	
Physical violence (58)		
Serious assault (50)		
Anxious/aggressive/disruptive patient (38)		
Death threats, including against family (34)		
Threats with/possession of any object/weapon (32)		
Sexual harassment (4)		
Taking hostages (3)		
Threats/violence against co-patients/relatives (21)	Violent acts towards others than staff	
Self-harm/suicide attempt (15)		
Staff aggression/violence (3)		
Active defence/intervention (70)	Ways the staff responded	Ways in which staff dealt with aggressive situations and the eventual outcome
Alarm/call for help (56)		
Call for police/fire brigade (31)		
Passive defence/de-escalation (30)		
Patient discharged from the ward (1)		
Mechanical restraint (69)	Ways in which the incident ended	
Delimiting/supervising (46)		
Calming down of patient/ebbing of situation (42)		
Removal from the ward (35)		
Enforced medication (31)		
Anaesthetising/rapid tranquillisation of the patient (5)		
Abscondment of patient (4)		

The figures in parentheses indicate the number of descriptions

#### Details of violent and aggressive acts and situations

Quite often, the CI descriptions included aggressive and violent acts towards property causing *material damage*, like kicking furniture, throwing a flowerpot or trashing a TV (*n* = 45). Sometimes, the aggressive behaviour stayed at that level, but at other times it escalated towards attacking the staff or other patients. Regarding *violent patient acts towards staff*, the most commonly reported types of aggressive behaviour were threats, provocations and general aggression (*n* = 89). The staff found it difficult to handle these sometimes-constant verbal threats and provocations and seemed to disagree on how to deal with these situations. Some were more accepting of rough language and felt that it goes with the territory of working on a psychiatric ward: something you have to accept and get used to. Others found it difficult and considered such behaviours very stressful psychologically, affecting their enthusiasm for going to work. Physical violence (*n* = 58) and serious assault (*n* = 50; the latter reserved for violence to the face, head or throat, or severe violence to the torso) were perceived as serious behaviours with the intention of inflicting pain or injuries. The severity was amplified given that this type of violence was often described as coming unannounced and catching staff off-guard*.* It could also happen when staff were administering medical injections or mechanically restraining violent patients.

In some cases, staff described needing to care for patients who were anxious, aggressive or disruptive (*n* = 38). One of the most negative and stressful types of incident involving aggression and violence was when patients issued death threats, including against family (*n* = 34). Such threats were described as triggering feelings of stress, fear and uneasiness. Aggression and violent behaviour became more serious if any kind of object or weapon was involved (*n* = 32). Sometimes, patients used a dinner knife, but any object that was made of glass or was sharp could be used as an object to threaten or injure people. In situations where patients used objects or weapons as a means of aggression, other patients were often described as being worried and upset.

In the same category were CI descriptions of four cases of sexual harassment, and situations where patients took hostages to get medicine or to get their own way in some other regard (*n* = 3). There were also incidents in which staff members felt cornered or restricted to a corner or a small room.

The category labelled *violent acts towards others than staff* includes aggression and violence targeted at relatives (*n* = 21)*.* The staff also had to help and protect other patients in addition to, in some cases, defending themselves. Under this category, we also included self-harm and suicide attempts (*n* = 15). Incidents involving staff being violent or abusive towards patients were reported in only three cases. Nevertheless, these could lead to negative consequences for the individual staff member; e.g. one member of staff was fired because of violence towards a patient.

#### Ways in which staff dealt with aggressive situations and the eventual outcome

The second main area includes descriptions of the different *ways the staff responded* to patient-related aggression. A common staff response to violent patients was some sort of active defence or intervention (*n* = 70), such as holding the patient so s/he could be put in mechanical restraints or given a medical injection. In many of the incidents, the staff felt that they had to alarm or call for help from other colleagues (*n* = 56). Sometimes the staff felt they had to call the police or the fire brigade (*n* = 31). An often-used alternative response to aggression was passive defence or de-escalation: an attempt to avoid triggering an already upset and aggressive patient (*n* = 30). The staff also described *the way in which the incident ended*. In the majority of the CI descriptions, some sort of active response was necessary to calm the situation down; e.g. the patient being strapped onto a bed (mechanical restraint, *n* = 69). A calmer outcome occurred in the incidents labelled delimiting/supervising (*n* = 46), where, for instance, the patient was told to go to his/her room, the patient calmed down or the situation ebbed (*n* = 42). Removal from the ward (*n* = 35) and enforced medication (*n* = 31) were two of the more active outcomes of aggression and violent behaviour, as were *anaesthetising/rapid tranquillisation of the patient* (*n* = 5). In four cases, according to the descriptions, the patient absconded.

### After the incident

I had problems sleeping and nightmares for some weeks after the incident.

The final unit of analysis focuses on what happened *after* the incidents. Here our analyses and categorisations established three main areas, eight categories and 31 subcategories (Table 3).

**Table 3:** Categorisation of staff descriptions of what happened *after* the 283 incidents

Subcategory (number of descriptions)	Category	Main area
Talking with colleagues(/superiors) (107)	Support to the staff involved	Support to staff and/or organisational and administrative measures taken
Professional guidance (9)		
Talking with family member/s (3)		
Contact with trade union (1)		
New working strategies (31)	Organisational and technical measures taken	
Measures regarding alarms/equipment (10)		
Temporary reinforcement of staff (8)		
Removing/restoring facilities/furniture (5)		
Staff moved/fired (4)		
Documentation/reporting to authorities (39)	Reporting the incident	
Reporting to the police (24)		
Increased stress levels/sleep problems (72)	Psychological effects on staff	Psychological and physical impact on staff
Fear (47)		
Increase in caution among staff (19)		
Reduced confidence in the staff (9)		
More tight-knit staff groups (5)		
Reflections on the incident (12)	Thoughts about the incident	
Powerless/exhausted/offended (11)		
Empathy for the patient (10)		
Bodily pain (29)	Physical damage to staff and their property	
Bodily harm (25)		
Broken glasses/personal belongings (2)		
Talking to the patient/co-patients (33)	Measures related to the patient/s and patient concerns	Consequences and concerns among patients and staff
Changing ways of working with patient (29)		
Patient transfer (15)		
Medical assessment/medication (11)		
Anxiety of co-patients (11)		
Discharge from ward (4)		
Staff dissatisfied with management (24)	Other effects described	
Trial (6)		
Continuous threats (4)		

The figures in parentheses indicate the number of descriptions

#### Support to staff and/or organisational and administrative measures taken

The most-common *support to the staff involved* in violent situations was talking with colleagues and, sometimes, with superiors (*n* = 107, of which 21 included superiors). This could be routine after certain incidents, or more improvised and informal if the staff members affected asked for a debriefing talk with colleagues and superiors. Other types of support were provided in the form of professional guidance (*n* = 9), conversations with family members (*n* = 3) or through contact with the union (*n* = 1). The second category in this first main area is *organisational and technical measures taken.* A number of the responses described new working strategies (*n* = 31), such as always having more than one staff member among the patients in the ward. In quite a number of the descriptions, there were problems with the alarm equipment, which induced stress and worry among the staff (*n* = 10). They asked for reassurances that colleagues would come if they pushed the alarm button*.* There were also reports of staff being fired or removed from the ward due to violent behaviour against patients (*n* = 4). Furthermore, our material showed two ways of *reporting the incident*, namely, documentation/reporting to authorities (*n* = 39) or reporting to the police (*n* = 24).

#### Psychological and physical impact on staff

The second main area focuses on the reported psychological and physical effects. The most commonly reported *psychological effects on the staff* were increased stress levels/sleep problems (*n* = 72) and fear (*n* = 47)*.* Another effect of aggressive and violent incidents on the staff was an increase in caution (*n* = 19). These CIs could lead to reduced confidence within the staff group (*n* = 9). By contrast, when the staff managed to deal with a violent incident successfully, that could lead to a more tight-knit staff group (*n* = 5).

The category *thoughts about the incident* included staff descriptions of their feelings and reflections about the incident (*n* = 12), such as questioning whether things could have been handled differently, feelings such as powerlessness, exhaustion and/or of being offended (*n* = 11), and empathy for the patient (*n* = 10). *Physical damage to staff and their property* was also reported: bodily pain (*n* = 29), bodily harm (*n* = 25) and broken glasses/personal belongings (*n* = 2). In some cases, staff members required surgical procedures, sometimes paired with lengthy sick leave.

#### Consequences and concerns among patients and staff

The third main area relating to events following the incident is comprised of two categories. In the first, *measures taken relating to the patient/s and patient concerns*, the two most-common subcategories were talking to the patient/co-patients (*n* = 33) and changing the ways of working with the patient (*n* = 29), such as avoiding being alone with him/her. The other subcategories in this category were patient transfer to some other ward/unit (*n* = 15), medical assessment/medication (*n* = 11), anxiety of co-patients (*n* = 11) and discharge from the ward (*n* = 4).

In the second category, *other effects described*, a number of the staff mentioned dissatisfaction with management (*n* = 24), such as experiencing a lack of support from superiors after the incident. Two further findings in this category were violent behaviour ending in a trial (*n* = 6), and continuous aggression and violence from certain patients during their stay on the ward (*n* = 4).

## Discussion

Taken as a whole, the 283 CI events in our material provide a rich pattern of the nature and degree of critical incidents of aggression and violence in a variety of psychiatric inpatient care settings. Staff reported serious physical violence as well as a number of different kinds of threatening situations. Violence was common in these settings, with nearly half of the responding staff reporting an experience of work-place violence during a six-month period [7].

With regard to factors of relevance before the incident, staff attributed violent incidents mainly to internal patient factors, e.g. psychiatric diagnoses, substance abuse or impulsive behaviour. The *internal patient factors* main area, which corresponds to the internal model of factors causing violent behaviour [11], comprises 347 separate descriptions across all subcategories. The *situational and relational factors* main area, which includes categories and subcategories relating to the situational/relational model [10, 13, 16, 17], comprises 196 descriptions across all subcategories. Many of the CI events were attributed to ongoing care issues, such as patients being denied medicine or being met with negative responses to requests. Staff rarely attributed problems to staff or organisational factors. The *external factors* main area, which includes categories and subcategories relating to the external model [14, 15], comprises only 14 descriptions across all subcategories.

Our study focuses on what the Safewards model labels as “flashpoints”: the events or social circumstances most likely to trigger a conflict or containment [22]. Giving a patient negative news or information, refusing a patient’s request or asking a patient to do, or stop doing, something are all examples of staff behaviour that can trigger violence [22, 36]. We can identify flashpoints in our study that relate to all six originating factors in the Safewards model. Although research has long shown that patient characteristics are just one of the causes of aggression in psychiatric care settings, attribution was heavily weighted to internal patient factors in our study. The same has been found in somatic care settings [45]. Only a few CI events (*n* = 39) were attributed to problems within the organisation or staff team. Our material does include some of the Safewards staff modifiers, such as staff anxiety and frustration, psychological understanding and checking routines. Originating factors such as stressors from outside the hospital, the regulatory framework and the physical environment are rarely found in our material, but there are some descriptions, e.g. being denied a driving licence and a big quarrel with the mother during home leave from the hospital.

The reports of what happened during the incident include many descriptions of verbal aggression, including death threats towards staff and their families, as well as very serious physical assaults, mostly by patients targeting staff. Few CIs involved sexual harassment or assault. Staff described 72 CI events where they used passive defence/de-escalation or where the patient calmed down and the situation ebbed. We chose to use the label de-escalation/passive defence because our interpretation of the text was that staff did not explicitly describe de-escalation techniques. From interviews [46] in the same settings, we know that staff seem to prefer to use the least-restrictive techniques when possible, but knowledge of de-escalation techniques seems to vary. Thus, despite the fact that de-escalation techniques were often used, awareness of the concept itself seems to be low. Although de-escalation is recommended [23, 30], it has been difficult to show that training decreases the number of violent incidents [47–49]. However, education can provide staff with the knowledge and confidence to manage aggression [47]. With de-escalation, the incident can also be viewed as a shared problem to be solved together with patients, thereby creating trust and safety [34, 50]. Training in the management of violence should be supported at an organisational level [49] to foster a culture of change towards a strength-based perspective and a more person-centred process of care [50, 51].

An important finding of our study is that staff reported that many incidents ended fairly quickly and with the staff in control of the situation. Nevertheless, 283 CI events described the use of restrictive practices such as alarming, contacting the police, using mechanical restraints, delimiting or medication. In only 20 cases were no restrictive practices reported at all. In five extreme cases, when communication with the patient and de-escalation were deemed impossible, rapid tranquillisers were used. In such cases, the patient may have used designer drugs or had delirium, leading staff to assess that the risk of serious injury was high.

The descriptions of CI events suggest that staff members were often alone with patients during critical situations. Even though there are always several staff members working on a ward, it appears that staff often work with patients in areas where other staff members cannot see or hear what is happening. Furthermore, alarms did not always work correctly or in all parts of the ward. This combination of working alone and non-functioning alarms may increase the risk of violent incidents having serious consequences. A lack of trained and experienced staff members was also described, which may aggravate potentially serious situations. This finding is supported by a recent study based on interviews with staff and ward managers from three of the clinics included in the present study [46].

Regarding the after-effects of the violent incidents, staff reported bodily harm and pain in 54 CI events, but psychological or emotional distress in 185 CI events. The latter effects included stress, fear, increased cautiousness and negative feelings. Similar findings have been reported in other studies [35]. Staff reported receiving support mainly by talking to each other or, more seldom, to their manager. Dissatisfaction with the management was sometimes reported. Active leadership, follow-up meetings and more formal professional guidance or debriefing were seldom described. Support is important for staff in psychiatric care environments, regardless of whether the need is expressed or not, because violence is often seen as “part of the job”, and staff can experience conflict between their duty to care for the patient and their duty to care for themselves [52]. Empathy for the patient was reported in some cases.

Our impression is that researchers and policymakers in psychiatry often give primary and secondary prevention more attention than tertiary prevention. Staff descriptions of the events following violent incidents have been somewhat neglected in earlier literature, although studies have reported that staff express a need for greater psychological support, either from their managers or in the form of professional guidance [46, 52]. The overall impression from our study is an absence of leadership after traumatic incidents, such as management generally taking a low profile in supporting staff after a violent event.

Guidelines from, for example, NICE [23] or the International Labour Office [30] pinpoint the importance of post-incident debriefing and formal reviews; however, the descriptions of CI events in our material indicate a lack of awareness or implementation of this part of the guidelines. Such interventions may be important, not least because studies indicate that 10 % or more of psychiatric hospital health workers suffer from PTSD [32, 33]. Moreover, staff may experience a subset of the symptoms of PTSD even if they do not always fulfil all the criteria for a formal diagnosis. The provision of professional support to staff, especially after a violent incident, is likely to be a key factor in improving preventive measures for reducing violence on wards. Staff who feel that they are not listened to or taken seriously, or who have their competence questioned, etc., probably perform sub-optimally in their roles, as well as experience negative feelings.

There has, however, been some concern about how effective debriefing actually is and whether it can, in fact, do harm. A 2003 review of the literature [53] on the effectiveness of single-session debriefing found no evidence that psychological debriefing prevented PTSD after a traumatic incident. It is important to remember that recovery from trauma is not something that happens quickly, but is rather a prolonged process that may involve multiple therapeutic sessions [54]. There is a need for further research on what kind of debriefing works in inpatient care and for whom [55]. Staff members in our study were disappointed that that their managers did not provide enough support and/or did not follow up with them after a violent incident. There is certainly a need for follow-up after incidents, but also for checking whether more support is needed after a week or two. In addition to post-incident trauma counselling, pre-placement personality evaluation of health workers prior to being assigned to psychiatric units may be beneficial [56].

### Strengths and limitations

The four concepts described by Fridlund [57] are relevant when it comes to evaluating our study: applicability, concordance, security and accuracy. Applicability refers to the participants and measurements chosen. Because our participants were from 10 different psychiatric clinics, they conformed well to the purpose of the study as well as ensured variety of data. In terms of measurements, CIT is a method that, if used correctly, has high credibility when studying actions and experiences [58]. Concordance refers to the validity of the design used with regard to the purpose of the study. Since our focus was on staff experiences of violence and aggression in their work in psychiatric inpatient care, the design was highly applicable. The concept of security refers to the trustworthiness of the study and is very important when using CIT since data can be categorised in more than one way. In our study, we processed the material several times, discussing subcategories, categories and main areas until we achieved total consensus. Finally, we ensured the accuracy or precision of the study and results obtained by never taking any findings for granted and by, at all times, using the staff’s written descriptions and direct quotations as the basis for the analysis.

We are of the opinion that the large number of cases analysed strengthens our study, creating a picture formed across many people and over a variety of settings. The large number of cases made it possible to quantify the frequency of the subcategories in our data, even though these numbers cannot be generalized. Limitations of the study include the splitting up of individual descriptions for categorisation, which risks the loss of a lengthy and potentially interesting flow within the text. In spite of the large number of cases, many of the descriptions were fairly short: it might have been possible to obtain more information. A weakness of our use of questionnaires is that we were unable to ask follow-up questions when the answers were incomplete. This was especially notable with regard to the strategies used by staff to solve the CIs.

The low response rate [443 responders (42%), of which only 181 reported CIs] may have influenced the results through selection bias and by providing the authors with less material to analyse.

Finally, the research group did not receive any demographic data for individual staff members. In retrospect, this lack of information concerning non-respondents is a limitation.

## Conclusions

Staff attributed many incidents of aggression and violence to internal patient factors. As a primary prevention effort, it is important to raise awareness that external factors (organisational, situational and relational) are also important causes of violence and may be easier to modify. A secondary prevention approach could be to improve competence among staff in the use of de-escalation techniques. The descriptions indicated that restrictive practices were more common than de-escalating practices when dealing with violent incidents. Awareness of de-escalation techniques appeared low, despite staff usually managing to take control of the situation quickly. Furthermore, staff mainly reported negative psychological effects after violent incidents, rather than bodily injuries, and either expressed their dissatisfaction with the lack of support from managers after an incident or did not mention management at all. An important tertiary prevention measure would be for management to follow up with staff regularly after violent incidents and to increase psychological support in such situations.

## Supplementary information

**Additional file 1.** Extended tables with quotes from the results section.

## Data Availability

The data contributing to these analyses are held in a secure database at Örebro University Hospital, Department of Occupational and Environmental Medicine (where one of the authors was working when the study was conducted). Researchers or clinicians seeking access to this data for academic non-commercial purposes are welcome to submit a request to the corresponding author (VPP).

## Abbreviations

**CI**: Critical Incident
**CIT**: Critical Incident Technique
**PTSD**: Post-traumatic stress disorder
