# Supplementary material for: Violence and aggression in psychiatric inpatient care in Sweden: a critical incident technique analysis of staff descriptions
Source: BMC Health Serv Res. 2020 Apr 26;20:362. doi: 10.1186/s12913-020-05239-w (PMC7184692; doi:10.1186/s12913-020-05239-w)
Supplement: Supplementary file 1 — Additional file 1. Extended tables with quotes from the results section. [file 12913_2020_5239_MOESM1_ESM.docx]

**Additional File**

**Extended Tables**

**Extended Table 1** Examples of staff descriptions referring to what happened *before* the 283 incidents. Figures in parentheses indicate the number of descriptions.

| **Description** | **Subcategory** | **Category** | **Main area** |
| --- | --- | --- | --- |
| Psychotic patient walking anxiously around corridor; knocking on office door often and seeking attention. | Psychiatric diagnosis/  symptoms/  conditions (106) | Patient traits and states | Internal patient factors |
| Try to communicate with patient who gets even more wound up. Patient starts hitting and speaking incoherently. | Disruptive/  violent/ impulsive behaviour (68) |  |  |
| The patient has a history of drug abuse, a constant paranoid readiness and solves problems with threats and violence. | Substance abuse (60) |  |  |
| Fetched patient from emergency reception. Fairly calm there. In the lift, patient looks at own reflection in mirror and becomes frightened. | Scared/ anxious/ frustrated/ suspicious (34) |  |  |
| Patient throwing tables and chairs in the room when two staff members are present. | Sudden threatening/ violent behaviour (34) |  |  |
| A colleague and I prevented an older man with dementia from taking down the decor in the ward. | Intellectual disability/ dementia/ brain injury (10) |  |  |
| Autistic patient was contact-seeking and came up to me. | Approaching staff suddenly and unpredictably (8) |  |  |
| Patient seeking asylum becomes threatening after talking to a doctor since patient doesn’t want to be discharged. | Foreign background (8) |  |  |
| Patient returns from leave. Has had a row with mother. Is very worried. I’m sitting for a while with patient. Patient finds razor blade. Starts cutting own hands. | Self-harm/ suicide attempt (7) |  |  |
| The patient showed inappropriate interest in my colleague; an "unhealthy fixation", virtually stalking her | Inappropriate sexual or misogynistic behaviour (5) |  |  |
| Female patient, multiple diagnoses, heavy addiction, sexually exploited. Becomes "crazy" if coffee is finished, if she doesn’t receive medicine, if she doesn’t find her stuff. | Historic sexual abuse of patient (1) |  |  |
| Not violent - but it doesn’t feel good that we should be caring for patients with serious somatic illnesses. | Serious somatic illness (1) |  |  |
| Patient with psychoses/drug abuse held in seclusion; Not given access to a room or a toilet because patient has trashed everything and smeared everything with excrement before. | Special and rare circumstances (5) |  |  |
| Too few medical consultations with doctor and doctor lacked interest in the patients. Verbal promises never kept. | Organisation problems/ inadequate handling (29) | Characteristics of organisation and staff | External factors |
| The staff had gathered in the office for report on a new patient; the patients were on their own in the dining room. | Change of shift/staff busy (7) |  |  |
| The atmosphere in the ward had escalated before the patient came. Little experience of this patient group in the ward. | Scared/ anxious/ inexperienced staff (6) |  |  |
| Planning with colleagues about how we should handle the situation | Staff preparing to intervene (1) |  |  |
| Patient not granted a driving license by Swedish Transport Agency due to substance abuse/addiction | Denied request/ unwanted news (58) | Unwanted decision or information | Situational and relational factors |
| Patient, who is being cared for in ward due to delusions, is to be medicated by injection but does not want to co-operate voluntarily. | Medicine denied or enforced (31) |  |  |
| Refusal of medication as well as poor insight of illness. Staff going to use coercive measures - injection. | Medication of patients (26) | Measures taken impacting the patient |  |
| Patient with substance abuse problems comes to ward; assessed by doctor. Doctor considers an LVM [compulsory care order re. substance abusers] decision is relevant. Leaves the ward and an angry patient. | Patient about to be admitted/ discharged (15) |  |  |
| Patient with no illness insight. Begins with doctor consultation with patient. Patient’s mood switches during conversation when told that depot injection will be given. | Patient in doctor or staff consultation (13) |  |  |
| Patient arrives in handcuffs with police. Directly into ward and a room. Under influence of drugs/alcohol (self-inflicted cut). Placed directly in belt restraints because of aggression | Police intervention (11) |  |  |
| The patient was monitored all the time. Two staff members present. | Patient supervised/ secluded (7) |  |  |
| I was called to help when administrating mechanical restraint. | Patient (about to be) mechanically restrained (6) |  |  |

The figures in parentheses indicate the number of descriptions

**Extended Table 2** Examples of staff descriptions referring to what happened *during* the 283 incidents. Figures in parentheses indicate the number of descriptions.

| **Description** | **Subcategory** | **Category** | **Main area** |
| --- | --- | --- | --- |
| Paranoid patient rips bit off a radiator and uses it to threaten the staff. We lock the patient in own room. Kicks the door, window, trashes the room. | Aggression against property (45) | Material damage | Details of violent and aggressive acts and situations |
| Patient threatens to break the door of drug room and talks about crushing the jaws of female staff. | Verbal threats/provocation/aggression (89) | Violent patient acts towards staff |  |
| The patient is a disruptive young woman who first kicks me in the stomach and then when I turn to go, kicks me in the back. | Physical violence (58) |  |  |
| I’m near the staff room when, completely unprovoked, a patient directs a punch at my face, hitting the right side of the bone on my nose. | Serious assault (50) |  |  |
| Patient being placed in safety straps. Turmoil ensues because the patient is strong and forcefully resists. | Anxious/aggressive/disruptive patient (38) |  |  |
| Patient kicked me in the groin and spat on my face and then threatened to kill me and my children. | Death threats, including against family (34) |  |  |
| When being informed about medicine, patient threatened staff with e.g. knife, lamp base and bottle neck (glass) in order to receive medication. | Threats with/possession of any object/weapon (32) |  |  |
| Patient is threatening, aggressive, makes accusations about religion and sex. Makes sexual advances and innuendos. | Sexual harassment (4) |  |  |
| The patient holds knife to staff member’s neck and threatens to injure her. | Taking hostages (3) |  |  |
| Patient hits another patient over the head with a vase. | Threats/violence against co-patients/relatives (21) | Violent acts towards others than staff |  |
| Patient cut his arm and threatened to infect me with hepatitis. | Self-harm/suicide attempt (15) |  |  |
| Another member of staff threatened a patient and was fired. | Staff aggression/violence (3) |  |  |
| It is decided that the patient must be restrained with straps. A struggle ensues. Staff sound the alarm, the police need to be called. Six members of staff have to wrestle down the patient and wait for the police. | Active defence/intervention (70) | Ways the staff responded | Ways in which staff dealt with aggressive situations and the eventual outcome |
| Psychotic patient screaming that he’s going to kill me; throwing chairs and ashtray at me. I alarm for other staff who get here quickly. | Alarm/call for help (56) |  |  |
| Young guy, high on internet drugs, was paranoid and violent. 14 people, including 2 police, were needed when restraining him with straps. | Call for police/fire brigade (31) |  |  |
| Patient takes a poker and threatens nurse I’m working with during the shift. I order all staff to back out of dining room and split up over the ward. | Passive defence/de-escalation (30) |  |  |
| Patient (with compulsory care order) had been violent. Arrives with police. Senior physician orders that restraints be used; no addictive drugs. After 36 hours, the patient was discharged from the ward. | Patient discharged from the ward (1) |  |  |
| Woman became violent; trashed TV room as she didn’t get medicine she wanted. Staff and police were alerted. Belt restraints used eventually and patient taken to another department. | Mechanical restraint (69) | Ways in which the incident ended |  |
| Patient is asked to go into the room to calm down, which he does. Staff check him regularly. | Delimiting/supervising (46) |  |  |
| When patient goes away, it becomes calm. | Calming down of patient/ebbing of situation (42) |  |  |
| Psychotic illness. Seemed to have improved but was paranoid. Lit fire in wastepaper bin in the corridor. We alerted police; fire brigade came. Police took patient to the hospital emergency room for a new assessment. | Removal from the ward (35) |  |  |
| Patient refuses medicine and continues to be threatening. Restrained with protective belt and involuntarily injected | Enforced medication (31) |  |  |
| Patient is under influence of internet drugs; patient is very aggressive and says he wants to kill. Patient taken by staff to ICU to be anaesthetised, staff | Anaesthetising/rapid tranquillisation of the patient (5) |  |  |
| The patient absconded during the trip. | Abscondment of patient (4) |  |  |

The figures in parentheses indicate the number of descriptions

**Extended Table 3** Examples of staff descriptions referring to what happened *after* the 283 incidents. Figures in parentheses indicate the number of descriptions.

| **Description** | **Subcategory** | **Category** | **Main area** |
| --- | --- | --- | --- |
| We talked to the staff affected so they felt OK going home after their work shift. | Talking with colleagues(/superiors) (107) | Support to the staff involved | Support to staff and/or organisational and administrative measures taken |
| If we wanted, we were allowed to request counselling from a psychologist from the occupational health service. | Professional guidance (9) |  |  |
| I wrote a non-conformance report, talked to colleagues, even talked at home with my husband. | Talking with family member/s (3) |  |  |
| Contacted by managers and trade union. | Contact with trade union (1) |  |  |
| I/we are more observant of patients' frame of mind and avoid being alone in the ward. | New working strategies (31) | Organisational and technical measures taken |  |
| Control of alarm sensors. However, two years later they are still not working as they should in the ward. | Measures regarding alarms/equipment (10) |  |  |
| This happened in the evening so when extra staff were called to the emergency room, it meant that the other departments were emptied of their staff. | Temporary reinforcement of staff (8) |  |  |
| The patient had rigged a noose using the sheets in the room. Removal of equipment | Removing/restoring facilities/furniture (5) |  |  |
| The patient began to threaten my colleague's family, which was the last straw for my colleague who dealt with the patient in an unauthorised manner. My colleague was immediately suspended and dismissed | Staff moved/fired (4) |  |  |
| I wrote a non-conformance report. I chose not to file a police report because it wouldn’t be the organisation; it would be me that would be the complainant. | Documentation/reporting to authorities (39 | Reporting the incident |  |
| I had a talk with my manager who helped me notify the police about the incident. | Reporting to the police (24) |  |  |
| I had a hard time sleeping and had nightmares for a few weeks afterwards. | Increased stress levels/sleep problems (72) | Psychological effects on staff | Psychological and physical impact on staff |
| Uneasy. Fear that the patient is hunting me down after release. | Fear (47) |  |  |
| Have thought more about having others (colleagues) with me during patient consultations. | Increase of caution among staff (19) |  |  |
| Felt insecure working with the staff for the rest of the shift. | Reduced confidence in the staff (9) |  |  |
| It was confirmed that the staff group it was able to handle a threatening situation in the ward. The group became more tight-knit. | More tight-knit staff groups (5) |  |  |
| For me, the dilemma is about whether you’re doing the right or wrong thing as a supervisor. | Reflections on the incident (12) | Thoughts about the incident |  |
| Powerlessness when you tell the doctor on intensive care about the patient’s condition but you are not listened to. | Powerless/exhausted/offended (11) |  |  |
| I had some sympathy for him . I didn’t bear any grudges. It must have been a terrible experience for him. He thought we were going to hurt him. | Empathy for the patient (10) |  |  |
| The police used tear gas on a patient. My eyes stung, I was coughing and had difficulty breathing. | Bodily pain (29) | Physical damage to staff and their property |  |
| The colleague who was beaten got a split eyebrow that needed stitches. | Bodily harm (25) |  |  |
| My glasses had to be thrown out; bruises and cartilage damage on my head/nose. | Broken glasses/personal belongings (2) |  |  |
| The patient calmed down and we sat for a while talking and listening to the patient; how he is and why he wants to go home. | Talking to the patient/co-patients (33) | Measures related to the patient/s and patient concerns | Consequences and concerns among patients and staff |
| I avoided being alone with this patient. | Changing ways of working with patient (29) |  |  |
| We relocated the patient to the Psychiatric Intensive Care Unit because there was a risk that the patient could be aggressive towards the staff (or other patients) again. | Patient transfer (15) |  |  |
| Medication and calming. Supportive conversation once the patient had calmed down. | Medical assessment/medication (11) |  |  |
| The other patients in the ward were quite terrified and anxious for two or three days after the incident. | Anxiety of co-patients (11) |  |  |
| The patient goes to Intensive Care to be anaesthetised. | Discharge from ward (4) |  |  |
| It concerned me and many others in the staff group that the staff member who reported the assault got so little support | Staff dissatisfied with management (24) | Other effects described |  |
| It was reported to the police, which resulted in a trial with most of the staff from our unit as the plaintiffs. | Trial (6) |  |  |
| Schizoaffective patient threatened us daily for about two months. Medication did not help. Several colleagues considered changing jobs, I did that for a while, returned later. | Continuous threats (4) |  |  |

The figures in parentheses indicate the number of descriptions
